# Meeting report: The 12^th^ European oral microbiology workshop (EOMW) in Stockholm, Sweden

**DOI:** 10.1080/21505594.2017.1376147

**Published:** 2017-11-10

**Authors:** Georgios N. Belibasakis, Michael A. Curtis, George Hajishengallis, Egija Zaura

**Affiliations:** a Department of Dental Medicine, Karolinska Institutet, Stockholm, Sweden; b Institute of Dentistry, Barts and the London School of Medicine and Dentistry, Queen Mary University of London, London, UK; c Department of Microbiology, School of Dental Medicine, University of Pennsylvania, Philadelphia, PA, USA; d Academic Centre for Dentistry Amsterdam (ACTA), University of Amsterdam and Vrije Universiteit, Amsterdam, The Netherlands

**Keywords:** oral microbiology, oral ecology, oral diseases, biofilms, microbiome

## Background

The EOMW is a forum that brings together junior and senior researchers with commitment, interest and track record in oral and general microbiology. It is a fruitful forum not only for exchanging knowledge and viewpoints, but also for developing new project ideas and building collaborations. Starting in 1984, it has since been held every three years and is hosted each time by a European dental institute. A historical list of all EOMWs so far is provided in the Table 1. The goals of the EOMW are to summarise the progress in the field over the past few years, to help shape current concepts in oral microbiology and to identify outstanding questions whilst setting approaches to address them. A number of such questions have indeed arisen and concepts have evolved since the last EOMW in 2014.^1–9^

**Table 1.** List of all EOMWs to date.

Year	Location/hosting city
2017	Stockholm, Sweden
2014	Aarhus, Denmark
2011	Utrecht, Netherlands
2008	Helsinki, Finland
2005	Thessaloniki, Greece
2002	Jena, Germany
1999	Brighton, United Kingdom
1996	Göteborg, Sweden
1993	Berne, Switzerland
1990	Cardiff, United Kingdom
1987	Aarhus, Denmark
1984	Nijmegen, Netherlands

**Figure 1.**
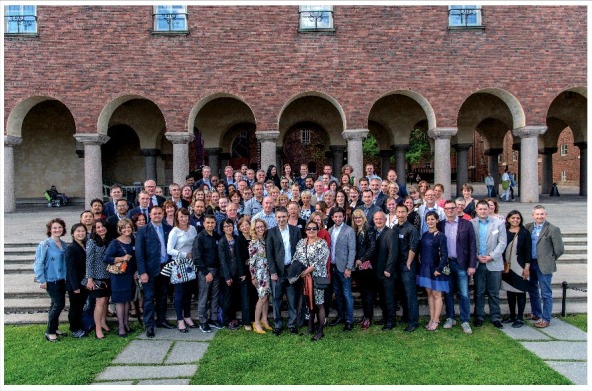
Group photograph of the 12^th^ EOMW participants in front of the Stockholm City Hall during the social event, on 26^th^ May 2017 (credit: Jack Mikrut).

The present meeting report aims to cover the scientific activities of the 12^th^ EOMW, which took place during 25^th^ – 28^th^ May 2017, at Lidingö island on the outskirts of Stockholm, Sweden. The hosting institute was Karolinska Institutet and was attended by 102 participants from all around Europe, but also Asia and the Americas. The social event took place on the evening of the 26^th^ May 2017 with a reception and guided tour at Stockholm City Hall (Fig. 1), the venue of the Nobel Prize banquet held on 10^th^ December each year, which were kindly hosted by the City of Stockholm and the Stockholm County Council. The scientific programme itself was structured in four themed oral sessions consisting of 25 oral presentations, including three keynote lectures, and a poster session consisting of 22 poster presentations. The four themed oral sessions were a) oral microbial ecology and the oral microbiome (chaired by Mike Curtis and George Belibasakis), b) dental caries and endodontic infections (chaired by Egija Zaura and Luis Chavez de Paz), c) periodontal and peri-implant diseases (chaired by Nagihan Bostanci and George Hajishengallis), and d) antimicrobial approaches (chaired by Gunnar Dahlen and Andrew Smith). The presentations from the oral sessions are elaborated further in this meeting report, whereas the corresponding abstracts are published in a 2017 supplement issue of the *Journal of Oral Microbiology*.^10^ Upon completion of the 12^th^ EOMW, the Satellite Symposium “Integrated diagnostics for oral infections” was held, within the frame of the EU Horizon2020 consortium DIAGORAS (www.diagoras.eu).^11^

## Oral microbial ecology and the oral microbiome

Following the opening address of the 12^th^ EOMW Chair George Belibasakis (Karolinska Institutet), Mike Curtis (Queen Mary University of London) opened the first session with his keynote lecture entitled “Oral Microbiology – progress, myths and evidence”. His presentation brought forward a number of significant advances in oral microbiology that have taken place over the past three years, such as the use of high throughput, non-cultural approaches for characterizing the oral microbiome, the advances in imaging methodologies used in characterizing oral biofilm architecture, and optimization of protocols to culture “not yet cultivatable” oral microorganisms. Mechanistic studies are also advancing through the use of ever more sophisticated and relevant *in vitro* and *in vivo* model of host-bacterial interactions systems, which enable a more comprehensive understanding of tissue homeostasis versus development of disease. Despite the progress, there is still increased need for appropriately designed and powered clinical studies, optimization of the application of high throughput analyses and improvement of the reliability of relevant model systems. The presentation led to an interactive discussion which focused upon several key issues, such as: to what extent has the recent application of high-throughput sequencing (HTS) of oral microbial populations enhanced our understanding of disease?; should our research focus shift from the “end stage organisms” in disease to analysis of the microbial populations represented by the “core microbiota” in order to understand disease pathogenesis; to what extent is the hereditability of periodontal disease a function of host germ line genetics versus parent to child transfer of an oral microbiome with the potential to lead to disease; why does the diversity of the periodontal microbiome increase in disease?; should treatment approaches focus on the host inflammatory/immune response or the bacteria.

Tsute (George) Chen (Forsyth Institute) elaborated further on principles introduced by the keynote speaker, by focusing on *in-silico* functional comparisons of genomic sequences of major human oral microbial species, in order to identify subsystems that are unique or missing among the genomes, and hence to obtain comprehensive functional insights into microbial genomics. Transposable elements were found in major putative periodontal pathogens, whereas genes involved in CRISPR were detected in most oral species. Nezar Al-hebshi (Temple University) presented evidence for the association of the oral bacteriome and mycobiome with oral squamous cell carcinoma. Several inflammatory bacterial attributes were enriched in the tumors, whereas mycobiome analysis revealed also a dysbiotic fungal community, dominated by *C. albicans*.

Jessica Neilands (Malmö University) investigated the effect of *Parvimonas micra* on the proteolytic activity of a multi-species bacterial consortium. Over longer periods of time, the gingipain activity of *P. gingivalis* was significantly higher when *P. micra* was present in the consortium, whereas shorter exposure of *P. gingivalis* alone to *P. micra* also resulted in increased gingipain activity. Susanne Bloch (University of Natural Reseources and Life Sciences of Vienna) presented data on *Tannerella forsythia*'s cell surface (S-) layer and its role in multispecies biofilm formation. The glycosylated S-layer did not affect the quantitative composition of the other species present in the biofilm, with the exception of *Campylobacter rectus*, but affected the localization of *T. forsythia* and its aggregation with *Porphyromonas gingivalis*. Mohamed Al-Haroni (UiT the Arctic University of Norway) discussed the conjugative elements of the Tn*916*/Tn*916*-like transposons, which contribute to the spread of antibiotic resistance genes between bacterial species. It was shown in selected oral streptococci and enterococci strains that both the excision rate of Tn*916* and its autonomous replication increased due to antimicrobial challenge, and loss of bacterial fitness was associated with multiple acquisitions of Tn*916*.

## Dental caries and endodontic infections

The next session dedicated to dental caries and endodontic infections was opened by the keynote lecture of Egija Zaura (University of Amsterdam and Vrije Universiteit Amsterdam), entitled ”OMICs in cariology and endodontology – what have we learned so far?”. She highlighted how OMICs techniques have conquered the field of oral microbiology during the last decade resulting in exponential growth of publications on the microbiomes of dental caries and root canal infections. She critically assessed whether these technological advances have provided us with meaningful insights to help clinicians choose appropriate treatment or prevention strategies. The questions that the cariology researchers would like to answer range from understanding fundamental biological processes, to caries biomarker selection in caries prediction/risk assessment, to the discovery of novel preventive strategies. Similar questions are being raised by endodontologists who would like to understand the biological processes behind clinical treatment outcomes, the effects of the debridement methodologies, the causality of the treatment failures and so on. Most current studies address the presence of the bacterial players – the microbiome – with the means of 16S rRNA gene amplicon sequencing. The majority of these studies, however, are small-scale, cross-sectional case-control studies that increase our basic knowledge on which bacteria may be found at the diseased state. Very few of the studies assess changes in microbiome in time.^12^ Longitudinal studies are crucial in answering the question regarding the causal role of oral microbial communities in the disease process and predicting disease risk. Functional approaches, such as looking at the entire gene pool within microbial communities – the metagenome – or at the actively transcribed processes – the metatranscriptome – have entered the field. Additionally, studying complex host and microbial activity products, assessed either as metabolome or proteome has become possible. These newer approaches are highly potent in providing knowledge for finding new clinical solutions: what finally matters is not the list of bacterial names, but what these communities are doing and why. The challenge for the researchers in the coming years will be to reach this next level and be able to integrate multiple complex datasets with clinical meta-data, paving the road to clinical breakthroughs.

Ingegerd Johansson (Umeå University) presented data on the salivary and tooth biofilm microbiota in caries and low-caries populations, using two sequencing platforms. The two sequencing platforms showed similar microbial patterns, whereas salivary microbiota discriminated caries-affected from caries-free adolescents with enumeration of five species (namely *Scardovia wiggsiae, Streptococcus mutans, Bifidobacterium longum, Leptotrichia sp*. HOT498, and *Selenomonas spp*) in caries-affected participants and one species thereof predicting 2-year caries increment. Anne Tanner (Forsyth Institute) compared the bacterial metatranscriptome of dentin caries to coronal caries and caries-free sites, using a next generation sequencing approach. Coronal caries sites exhibited with higher gene expression than caries-free sites. Dentin caries had greater number of over-represented activities from Gene Ontology (GO) terms compared with health or coronal caries, which were associated with sugar metabolism. *Scardovia wiggsiae* also appeared to be a major player in lesions advanced into dentin. Hence, this species appears to be a novel putative pathogen associated with dental caries.^13,14^ Thuy Do (University of Leeds) compared the bacterial metatranscriptome of root caries to sound root surface biofilms using an RNA-sequencing approach. Differential analysis indicated unique gene expression profiles in carious and sound root surface biofilms.

Moving more towards the field of endodontics, Ilona Persoon (University of Amsterdam and Vrije Universiteit Amsterdam) assessed the complex microbiome of the root canal system using next generation sequencing. Most frequently identified genera were *Prevotella, Lactobacillus, Actinomyces, Fusobacterium* and *Atopobium*, whereas more than half of the teeth were also positive for fungi, and those exhibited a significantly different bacteriome than the fungi-negative teeth. Finally, Luis Chavez de Paz (Karolinska Institutet) discussed how the inclusion of the biofilm concept in endodontics has enhanced our understanding of root canal and periapical infections, particularly the realization that microbial biofilms are formed inside the root canal. He critically highlighted that main concepts underlying the biological processes of root canal biofilms remain unclear, and presented a hypothesis where the endodontic biofilm may be considered as an extension of dental plaque.

## Periodontal and peri-implant diseases

The third session was dedicated to periodontal and peri-implant diseases. It opened with the keynote lecture of George Hajishengallis (University of Pennsylvania), entitled “Dysbiosis and inflammation in periodontitis: Synergism and implications for treatment”. He critically discussed that recent microbiome and host response mechanistic studies indicate that periodontitis is not a classical bacterial infection but rather results from indigenous polymicrobial synergy and dysbiosis due to breakdown in host-microbe homeostasis. He highlighted how the disease can develop in susceptible hosts by a polymicrobial community, in which different members have distinct functional roles that, yet, converge synergistically. He particularly discussed the role of keystone pathogens. The concept of keystone pathogens is one where their impact has community-wide significance and is disproportionate of their relative abundance. They can subvert the host response in ways that uncouple inflammation from bactericidal activity, thereby disrupting homeostasis and leading to the emergence of a dysbiotic microbiota, wherein inflammophilic pathobionts exacerbate the inflammatory response causing tissue destruction. Since inflammatory tissue breakdown products serve as nutrients for the persistence of dysbiotic microbial communities, it becomes evident that inflammation and dysbiosis reciprocally reinforce each other, accounting – at least in part – for the chronicity of periodontitis.

The next presentation by Asil Alsam (Queen Mary University of London) provided evidence that specific pathogen free (SPF) laboratory mice have a simpler microbiolome composition compared to wild wood mice (*Apodemus sylvaticus*), which displayed a qualitatively greater and quantitatively more complex oral microbiome. In this “natural” periodontitis mouse model, periodontal bone loss was also significantly elevated compared to SPF mice, which may account for differences in the microbiome. Abish Stephen (Queen Mary University of London) investigated the association between oral malodour and periodontal microbiota on the tongue of individuals with periodontal diseases. Disease-associated changes were observed in the distribution of oligotypes of health and periodontitis-associated genera between the oral niches, particularly on the tongue.

Anders Johansson (Umeå University) discussed variations in the expression of *Aggregatibacter actinomycetemcomitans*‘ leukotoxin. Except for the well-known JP2-genotype, another highly leukotoxic genotype exists with a full-length leukotoxin promoter, an identical arbitrary primed (AP)-PCR-pattern as the JP2-genotype, an intact *cagE* gene and serotype b classification.^15^ This *A. actinomycetemcomitans* subgroup is highly prevalent in samples of subgingival plaque collected from periodontal pockets of young periodontitis patients.^16^ Mark Lindholm (Umeå University) followed up with a mechanistic study aiming to investigate how outer membrane proteins (Omp) contribute to serum resistance of *A. actinomycetemcomitans* and *Aggregatibacter aphrophilus*, using genetically modified knock-out mutants. Whereas Omp100 of (*A. actinomycetemcomitans*) appeared not to contribute to serum resistance, deletion of o*mpA* in both species significantly reduced their survival in human serum. Riikka Ihalin (University of Turku) discussed secreted or outer membrane proteins of *A. actinomycetemcomitans* that interact with host cytokines, and elaborated particularly on the structure of the bacterial interleukin receptor I (BilRI) and secretin channel HofQ. Tuuli Ahlstrand (University of Turku) characterized the binding of IL-8 to HofQ, proposing a mechanism of internalization of IL-8 into the bacterial cells.

Graham Stafford (University of Sheffield) brought forward the often-neglected role of glycoproteins on the host-pathogen interface, particularly since bacteria have evolved enzymes to access those for their benefit. He focused on a range of novel CaZymes from periodontal pathogens discussing their role in host-bacteria interactions and biofilm formation, and their basic biochemical and structural biology. Karina Persson (Umeå University) presented data on the structure and assembly mechanisms of *P. gingivalis* Mfa fimbriae, using X-ray crystallography and discussed which structural part of the protein could function as a donor strand upon fimbrial polymerization. Rory Watt (University of Hong Kong) compared the distributions of phylogroup 1 and 2 oral treponemes in subjects with various periodontal health conditions, via sequence analysis of *pyrH*, a highly-conserved treponeme ‘housekeeping’ gene. He concluded that both healthy and periodontally-diseased subjects harbor multiple genetic lineages of the same treponeme phylotype within a given subgingival niche. Heli Jäsberg presented data on the effect of orally administered probiotics on the salivary levels of matrix metalloproteinases (MMP)-8, MMP-9 and their tissue Inhibitor of metalloproteinases (TIMP)-1 in healthy adults. She found increased MMP-9 and decreased TIMP-1 levels in saliva, indicating that probiotics have local oral immunomodulatory effects. Pirjo Pärnänen (University of Helsinki) presented data on a cell wall protein of the opportunistic yeast *Candida glabrata*, which could activate proMMP-8.

Dierdre Devine (University of Leeds) presented data on the association between the subgingival microbiome, periodontitis and rheumatoid arthritis, using next generation sequencing. Rheumatoid arthritis displayed high proportions of *Bacteroidetes*, with the relative abundance of *P. gingivalis* being also high in periodontitis and rheumatoid arthritis. Youngnim Choi (Seoul National University) investigated the microbiota of periodontal tissue lesions in comparison with the corresponding biofilms, using 16S rRNA pyrosequencing analysis. There were no significant differences in species richness or diversities between tissues and biofilms, but inter-subject variation within periodontal tissues was smaller than in biofilms, with increased proportions of *Fusobacterium nucleatum* and *P. gingivalis* within tissues. Danae Apatzidou (Aristotle University of Thessaloniki) compared the microbiomes of peri-implantitis and healthy dental sites in patients with a history of periodontitis, using a 16S-rRNA gene sequencing platform. Microbiome diversity was lower at diseased compared to healthy sites, but taxa abundance was higher in disease.

## Antimicrobial approaches

The fourth and last session of the workshop focused on antimicrobial approaches. Andrew Smith (University of Glasgow) presented collated data on the antimicrobial susceptibility of isolates from acute dento-alveolar infections over a 27-year period in Glasgow, with no discernable increase in resistance patterns observed over this period of analysis. He further discussed the importance of diagnostic oral microbiology, concluding that it matters in guiding antimicrobial stewardship programs, but more collaborative initiatives on processing methods and routine susceptibility data would help overcome limited data from single centers. Gunnar Dahlen (University of Gothenburg) discussed data from the Swedish strategic program against antibiotic resistance (STRAMA), noticing an overall decrease in antibiotic prescription over the last 10–15 years. Reportedly, total prescriptions in dentistry decreased from 35 to 27 per 1000 inhabitants between years 2007 and 2016, respectively, whereas the prescription rate for amoxicillin also decreased 30% between 2012 and 2014, probably due to revised prophylaxis guidelines. It was also noted that there are still significant differences in the prescription patterns between dentists in greater cities and rural provinces.

Moving towards novel antimicrobials, Mohammed Al-Zubidi (University of Sheffield) discussed lytic bacteriophages targeting *Enterococcus faecalis*, as a biological approach to handle root-canal infections. Lytic bacteriophages belonging to the *Siphoviridae* family proved to be specific to *E. faecalis*, and were able to eradicate such biofilms from abiotic surfaces. Georg Conrads (RWTH Aachen University Hospital) introduced a new design for bioactive coatings based on aqueous nanogels. Isoeugenol-modified nanogels exhibited superior antibacterial properties towards well-established oral pathogens, and were proposed as a suitable device for bio-interfaces. Finally, Monika Astasov-Frauenhoffer (University of Basel) investigated a nanostructured titanium surface for bacterial-colonization capacity, and it was shown to prevent vital bacterial adhesion in comparison to control surfaces.

## Conclusion

Overall, the 12^th^ EOMW has been a successful forum where a rich content of diverse oral microbiology topics were discussed. An important conclusion is that high-throughput technologies have helped us explore in depth the oral microbiome and paved new ways for studying the microbial metabolome, as well as the microbial or host proteomes. Culturing and studying in more detail previously unculturable species is also becoming increasingly feasible. It has also become evident that inflammophilic pathobionts orchestrate and reinfornce the reciprocal relationship between inflammation and dysbiosis. While the scientific community has been successful in characterizing individual microbial species, broader microbial communities, or dysbiotic host responses, we also begin to understand that conversion from health to disease entails a collective switch of the biological functions of the microbial communities. An important challenge is to understand better these functions by using combined omics approaches, to decipher the role of individual species in orchestrating them, and to define factors that are driving community shifts towards a dysbiotic state. Hence, we do feel that sufficient progress has been made since the last EOMW, with refinement of our current concepts and identification of new challenges to address and to re-evaluate during the next EOMW. The torch is now passed from Karolinska Institutet to UiT The Arctic University of Norway, which will host the 13^th^ EOMW in Tromsø, northern Norway, in 2020.
